# Taxonomic insights and new geographic records of *Metzgeriaepiphylla* A. Evans (Metzgeriales, Metzgeriaceae): A re-assessment based on discoveries in Shennongjia National Park, China

**DOI:** 10.3897/BDJ.12.e139010

**Published:** 2024-11-22

**Authors:** Wei Han, Qiang He, Youzhi Li, Jiaojiao Jin, Yu Jia

**Affiliations:** 1 School of Resources & Chemical Engineering, Sanming University, Sanming, China School of Resources & Chemical Engineering, Sanming University Sanming China; 2 State Key Laboratory of Plant Diversity and Specialty Crops/State Key Laboratory of Systematic and Evolutionary Botany, Institute of Botany, Chinese Academy of Sciences, Beijing, China State Key Laboratory of Plant Diversity and Specialty Crops/State Key Laboratory of Systematic and Evolutionary Botany, Institute of Botany, Chinese Academy of Sciences Beijing China; 3 Shennongjia National Park Administration/Key Laboratory of Conservation Biology of Golden monkey in Shennongjia of Hubei Province, Shennongjia, China Shennongjia National Park Administration/Key Laboratory of Conservation Biology of Golden monkey in Shennongjia of Hubei Province Shennongjia China

**Keywords:** Marchantiophyta, *Metzgeria*, new record, Shennongjia National Park

## Abstract

**Background:**

*Metzgeria*, a genus established in 1818, has been the subject of various taxonomic studies, with revisions leading to the recognition of several genera within the Metzgeriaceae family. Recent research indicates that *Metzgeria* constitutes over 98% of the family’s species, with broad global distribution.

**New information:**

During a bryophyte survey in Shennongjia National Park, *Metzgeriaepiphylla* A. Evans was newly recorded in China, expanding the known distribution of this species beyond its previously reported locations in the South Atlantic and South Pacific regions. This represents the first report of *M.epiphylla* in East Asia, specifically in evergreen broadleaf forest habitats. The discovery contributes new insights into the species' morphology, including variability in thallus shape and reproductive structures, distinguishing it from closely-related taxa. A key to species of *Metzgeria* from Asia is also given.

## Introduction

The genus *Metzgeria*, established in 1818 by Raddi (1818), belongs to the family Metzgeriaceae and was traditionally reported as a monogeneric family. Early works by Lindberg (1877) documented 11 species within *Metzgeria*, while subsequent research by Land (1911) focused on spore morphology to explore species differentiation. More recent taxonomic studies, such as those of Kuwahara (1966), Kuwahara (1973) and Kuwahara (1978a), have divided the family Metzgeriaceae into four genera: *Apometzgeria*, *Austrometzgeria*, *Metzgeria* and *Steereella*. Current molecular and morphological analyses (Furuki and Dalton 2013, Söderström et al. 2016) recognise three genera in the family—*Metzgeria*, *Steereella* and *Vandiemenia*. Amongst them, *Metzgeria* accounts for over 98% of the species. It exhibits a wide geographical distribution, with approximately 50 species found in tropical South America, seven in North America, six in Europe, nine in Asia, eight in Africa, 18 in Oceania and the Pacific and two in polar regions (Frey and Michael 2009). In recent years, Bechteler et al. (2021) comprehensively analysed the phylogenetic relationships, divergence times and ancestral distribution ranges of *Metzgeria* using molecular data, which not only deepened our understanding of the evolutionary and biogeographical history of the genus, but also provided a significant reference framework for further studies on other epiphytic bryophytes.

In China, research on Metzgeriaceae has been ongoing, with earlier studies documenting two genera and several species across the south-western regions. Zhang (1991) reported two genera and seven species, while later research by Gao and Wu (2010) expanded this to 13 species. The most comprehensive revision was conducted by So (2003), who retained 10 species within the genus *Metzgeria*, based on their morphological characteristics and distribution patterns in Asia. Zheng (2024) investigated the unavailability and typification of type specimens of *Metzgeria* taxa described by Yukinobu Kuwahara, particularly in NICH and proposed new typifications based on newly-located specimens to maintain nomenclatural stability. These findings highlight the significant diversity and complexity within the genus, particularly in regions like Asia where taxonomic revisions have clarified species boundaries.

Recent fieldwork in Shennongjia National Park, Hubei Province, China, revealed the presence of *Metzgeriaepiphylla* A. Evans, a species previously known only from the South Atlantic and South Pacific regions. This species, first described with its type locality in Chile (Evans 1923), has been reported from locations such as Tristan da Cunha, Papua New Guinea and New Zealand (Kuwahara 1978b, Piippo 1991). The discovery of *M.epiphylla* in China marks the first record of this species in East Asia, further extending its known range.

## Materials and methods

Two specimens of *M.epiphylla* were collected during field surveys conducted in Shennongjia National Park, Hubei Province, China, in May and September 2023 and deposited in the Herbarium (PE), Institute of Botany, the Chinese Academy of Sciences, Beijing. Collected specimens were identified by Y. Jia and Q. He from the Institute of Botany, Chinese Academy of Sciences. Photographs were taken using Leica DM 4000 B LED microscope (Leica Microsystems, Wetzlar, Germany). In addition to the collected specimens, one specimen of *Metzgeriaconsanguinea* Schiffn (collected on 5 May 2009, with Collection Number 2576, by Dr. Ningning Yu from the Institute of Botany, Chinese Academy of Sciences, Beijing, from a branch at altitudes of 879-886 m in Huangjing Town, Gulin County, Luzhou City, Sichuan Province, China, at coordinates 28°17’48″N and 105°46’07″E) was also examined to facilitate the differentiation between the two species.

## Taxon treatments

### Metzgeria epiphylla

A. Evans

391E47A8-925E-573B-8C09-9D970639FBEA

#### Materials

- **Type status:** Other material. **Occurrence:** catalogNumber: PE02162938; recordNumber: 4664; recordedBy: W. Han; occurrenceID: 7F65BDC7-D662-5D03-B467-A8B609B1FC94; **Taxon:** class: Jungermanniopsida; order: Metzgeriales; family: Metzgeriaceae; **Location:** continent: Asia; country: China; countryCode: China/CN; stateProvince: Hubei; county: Shennongjia Forestry District; municipality: Muyu Town; locality: Shennong Valley; verbatimLocality: Next to the boardwalk; verbatimElevation: 2820 m; verbatimLatitude: 31°26'19.47″N; verbatimLongitude: 110°16'52.0″E; **Identification:** identifiedBy: Y. Jia and Q. He; **Event:** year: 2023; month: 9; day: 18; habitat: On the trunks of *Abiesfargesii* Franch. in the forest
- **Type status:** Other material. **Occurrence:** catalogNumber: PE02154417; recordNumber: 948; recordedBy: Z.Q. Yi and J. Wu; occurrenceID: 636DFB31-73E3-5D9A-9ADC-1AC5AEF439C2; **Taxon:** class: Jungermanniopsida; order: Metzgeriales; family: Metzgeriaceae; **Location:** continent: Asia; country: China; countryCode: China/CN; stateProvince: Hubei; county: Shennongjia Forestry District; municipality: Muyu Town; locality: Banbi Rock; verbatimLocality: In the gully; verbatimElevation: 2590 m; verbatimLatitude: 31°27'26.35″N; verbatimLongitude: 110°13'41.44″E; **Identification:** identifiedBy: Y. Jia and Q. He; **Event:** year: 2023; month: 5; day: 14; habitat: On the branches

#### Description

**Figure 1.**
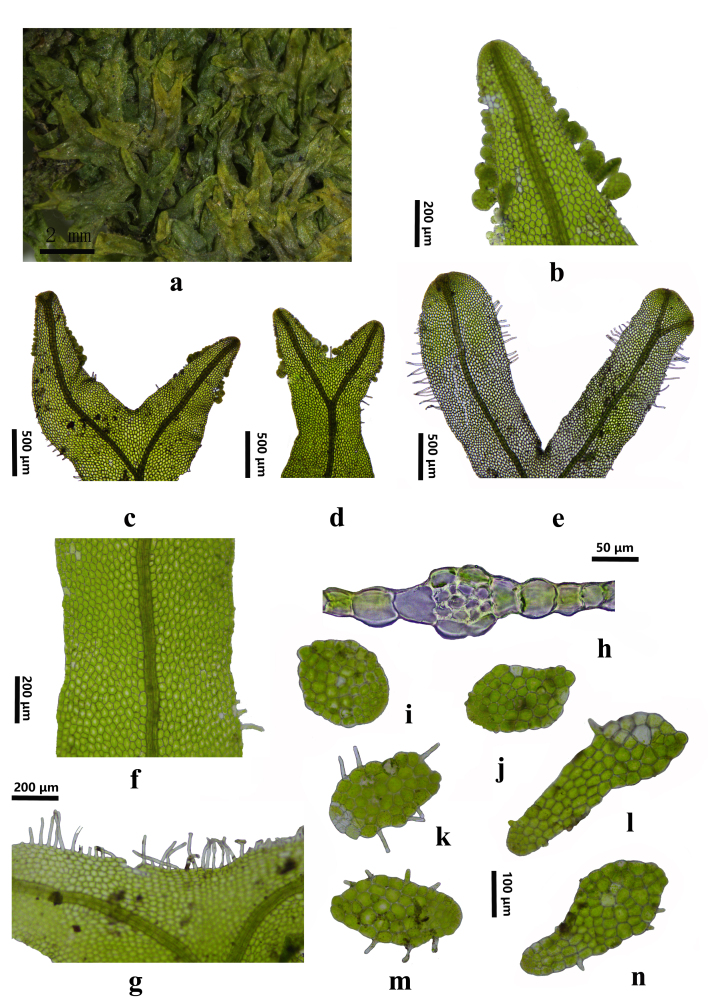
*Metzgeriaepiphylla* A. Evans. **a** Plants; **b-d** Tapered apex of thallus; **e** Obtuse apex of thallus; **f** Parts of thallus; **g** Marginal hairs; **h** Cross-Section of midrib; **i-n** Gemma. All from *HAN Wei 4664*.

The plant is yellow-green to pale green, lacking blue spots when dry, forming loosely interwoven mats attached to the substrate (Fig. 1a). The thallus is prostrate, irregularly forked, flat or slightly convex, with adventitious branches arising from the midrib on the ventral side. Mature thalli are 0.6-1.2 mm wide, occasionally up to 1.5 mm and 1-1.2 cm long (Fig. 1f). Branches are spaced 1-2.5 mm apart and exhibit two apex forms: one rounded and blunt (Fig. 1b-d), the other tapering into a short, sharp point (Fig. 1e). The midrib in cross-section has two layers of elliptical epidermal cells on both dorsal and ventral surfaces, measuring 25 × 15 μm, with three layers of 10 medullary cells, which are round to elliptical, thin- or thick-walled and 15-20 μm in diameter (Fig. 1h). The thallus is 16-22 cells wide from the midrib to the margin, with central cells approximately 40 μm in diameter and marginal cells slightly smaller at 25 μm (Fig. 1f).

Sparse spiny hairs occur along the leaf margins, usually solitary, measuring 0.1-0.2 mm long and 8-10 μm wide (Fig. 1g). Numerous gemmae are found along the edges or near the margins of narrow, specialised branches. These gemmae are variable in shape, ranging from round, elliptical, to elongated rod-like forms, flat or slightly convex, with short spiny hairs along the edges or ventral surface (Fig. 1i-n).

The species is dioecious. Male branches are nearly spherical, smooth, measuring 0.3-0.4 mm long and 0.25-0.35 mm wide. The female perichaetial leaves are broadly obovate, 0.3-0.35 mm in length and width, with spiny hairs on the edges and ventral surface. The capsule is brown, oval-shaped, typically ranging from 0.5-0.6 mm in length and 0.35-0.4 mm in width and the valves, when spread out, measure 0.6-0.75 × 0.2-0.25 mm. The calyptra measures approximately 1 mm in length and 0.45 mm in width, with abundant hairs above the middle and sparse, scattered hairs below. The spores are pale yellowish-brown and finely punctulate, with a diameter of 16-18 μm. Elaters are 0.3-0.4 mm long, 6 μm wide at the middle and bear a single broad spiral band extending the entire length. Gemmae are sometimes abundant, arising from more or less narrowed and specialised branches with limited growth, positioned either marginally or submarginally and dorsally and are orbicular to oval in shape, either flat or slightly convex, with a few short marginal hairs that are slightly displaced to the concave surface (Evans 1923).

#### Distribution

China (present study), Chile (Evans 1923), Tristan da Cunha, Papua New Guinea and New Zealand (Kuwahara 1978b, Piippo 1991).

#### Ecology

**Figure 2.**
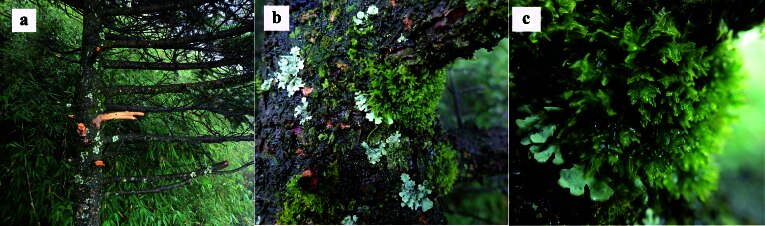
*Metzgeriaepiphylla* A. Evans in wild. **a** Habitat；**b, c** Populations. All from *HAN Wei 4664*.

This discovery represents a significant extension of the known distribution of *M.epiphylla*, confirming its presence in China, specifically in the evergreen broadleaf forests of Shennongjia National Park, where it inhabits tree trunks, branches and leaves at altitudes ranging from 1400 to 2900 m (Fig. 2a-c). Evans (1923) documented that plants of *M.epiphylla* preferred living leaves as a habitat, although they occasionally grew on bark. However, Piippo (1991) further described that the species occurred in montane rainforests of the Huon Peninsula and was specifically found on tree trunks. This habitat description closely aligned with the location where we discovered *M.epiphylla* in the present study.

#### Taxon discussion

During the bryophyte survey in Shennongjia National Park, two specimens of *M.epiphylla* were collected. Both specimens were identified, based on their morphological characteristics and matched the diagnostic features of *M.epiphylla*, as described in the original species description from South America (Evans 1923). The thallus was pale green, loosely interwoven and epiphytic, with irregularly forked branching patterns. Thalli measured 0.6-1.2 mm in width, occasionally reaching up to 1.5 mm, which differs from Evans (1923)'s original description, where mature thalli rarely exceeded 1 mm. Kuwahara (1978b) observed various forms of the thallus in *M.epiphylla*, although he did not provide specific measurements. This suggests that the thallus of the species exhibits a certain degree of variability, likely due to environmental factors or other sources of plasticity.

Further microscopic examination revealed detailed structural features consistent with *M.epiphylla*. The midrib consisted of two layers of epidermal cells, with three layers of medullary cells. The midrib cells were elliptical and measured 25 × 15 μm, while the medullary cells were 15-20 μm in diameter. Leaf edges had sparse spiny hairs measuring 0.1-0.2 mm in length. Spores were light brown, with a finely warty surface and measured 16-18 μm in diameter. The elaters exhibited single spiral thickenings, measuring 0.3-0.4 mm in length and approximately 6 μm in width. These characteristics confirm the identity of *M.epiphylla* and distinguish it from closely-related species within the genus.

## Identification Keys

### Key to species of *Metzgeria* from Asia

**Table:** 

1	Thallus apex with only one type, rounded	*M.decipiens*
–	Thallus apex with two types, conical and rounded	2
2	Male thallus apex conical, female thallus apex rounded	*M.acuta*
–	Both male and female thalli have dimorphic apices, without sexual dimorphism in branch structure	3
3	Thallus apex gradually tapering into a longer, narrow shape	4
–	Thallus apex sharply tapering into a short, blunt shape	9
4	Thallus large, strongly convex dorsally, with protruding midrib epidermal cells	*M.macrocellulosa*
–	Thallus medium or small, flat or slightly convex, midrib epidermal cells not protruding	5
5	Thallus with blue spots on the dorsal surface when dry	6
–	Thallus without blue spots on the surface when dry	7
6	Midrib cross-section with 4/4 dorsal/ventral epidermal cells	*M.fruticulosa*
–	Midrib cross-section with 2/2-3 dorsal/ventral epidermal cells	*M.darjeelingensis*
7	Numerous gemmae, capsule wall with thickened middle layer	*M.temperata*
–	Few gemmae, capsule wall without a thickened middle layer	8
8	Ventral surface of the midrib without rhizoids or spiny hairs	*M.sinensis*
–	Ventral surface of the midrib with rhizoids and spiny hairs	*M.consanguinea*
9	Thallus midrib epidermal cells 3-4 in number	*M.harae*
–	Thallus midrib epidermal cells 2/2-3 in number	10
10	Spore diameter exceeds 30 μm	*M.macrospora*
–	Spore diameter does not exceed 30 μm	*M.epiphylla*

## Discussion

The identification of *Metzgeriaepiphylla* in Shennongjia National Park represents a significant extension of this species’ known geographical range. Previously recorded only in regions such as South America, the South Atlantic and the South Pacific (Frey and Michael 2009), this discovery in China marks its first report in East Asia. The habitat of *M.epiphylla* in Shennongjia National Park is consistent with its typical occurrence in evergreen broadleaf forests, indicating its adaptability to montane environments in Asia. This finding not only contributes to the understanding of the distribution patterns of *Metzgeria* species, but also highlights the potential for undiscovered bryophyte diversity in Asia, particularly in montane evergreen forests.

The classification of *M.epiphylla* is a challenging endeavour due to its morphological diversity, particularly the presence of two distinct forms of leaf tips within various species. Bechteler et al. (2021) identified that several morpho-species of *Metzgeria*, including *M.furcata* (L.) Corda and *M.leptoneura* Spruce, exhibit polyphyletic characteristics. However, their study did not include *M.epiphylla*, leaving its specific phylogenetic relationships unexplored. Additionally, we have not yet discovered superior morphological traits for distinguishing the species, necessitating the use of the traits currently available in this study. The dimorphism, characterised by blunt and acutely pointed leaf tips, is a notable trait in several species distributed primarily in the tropical regions of South America. In Asia, similar dimorphic patterns can be observed in species such as *Metzgeriaacuta*, *M.consanguinea* and *M.darjeelingensis*, *M.fruticulosa*, *M.harae*, *M.macrocellulosa*, *M.macrospora*, *M.sinensis* and *M.temperata*. Historically, many of these species, except *M.temperata*, have been synonymised under *M.consanguinea* without substantive justification (So 2003), prompting a re-evaluation of whether this amalgamation accurately reflects the morphological and ecological distinctions amongst these taxa. Morphological investigations reveal significant differences amongst these species, despite apparent superficial similarities. For instance, *M.acuta* displays conical leaves uniquely in male specimens, whereas female counterparts have blunt leaf tips. The absence of conical leaf edges in the original description of *M.consanguinea* and the lack of distinctive sporophytes in various specimens further complicate its classification. These findings highlight the importance of a thorough assessment of mature morphological structures rather than relying solely on leaf tip characteristics for taxonomic decision-making.

**Figure 3.**
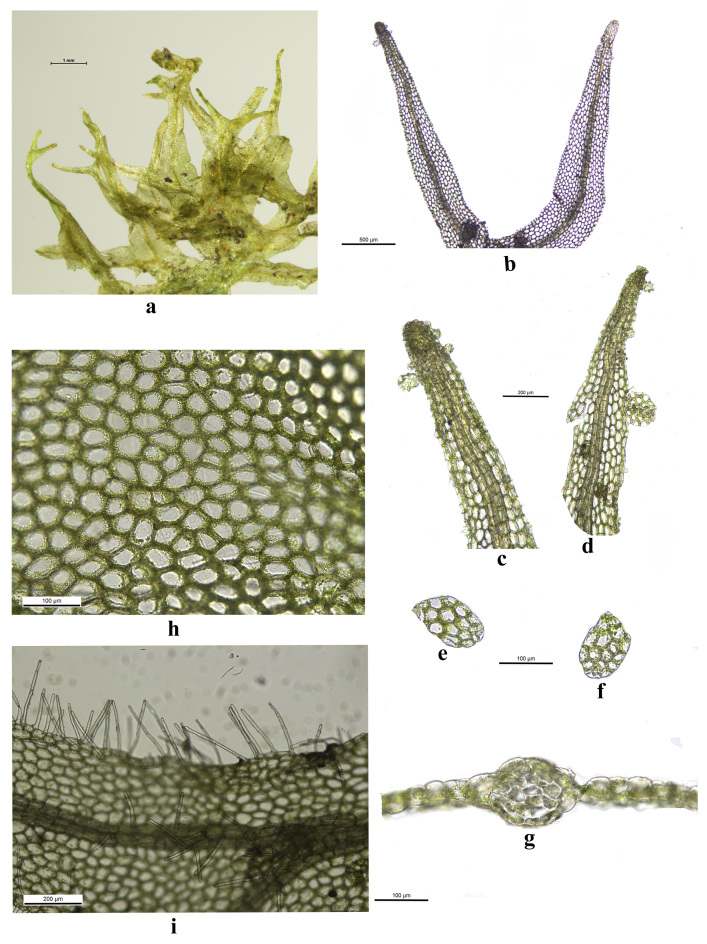
*Metzgeriaconsanguinea* Schiffn. **a** Plants; **b-d** Tapered apex of thallus; **e-f** Gemma; **g** Cross-Section of midrib; **h** Parts of thallus cells; **i** Hairs on the marginal and midrib of thallus. All from specimen *YU Ningning 2576*.

The historical classification practices by So (2002) have largely attempted to consolidate *M.epiphylla* within *M.consanguinea* based on leaf morphology and epidermal cell structures. However, this consolidation overlooks critical distinguishing features, such as sporophyte forms and leaf cell anatomy. For instance, Piippo (1991) noted that, while some vegetative thalli and all gemmiferous branches of *M.epiphylla* exhibit a more or less tapered morphology, its tapering is never as abrupt as that seen in *M.consanguinea*. Upon examining the specimen of *M.consanguinea*, we observed a distinctively tapered leaf tip (Fig. 3a-d), a feature markedly different from that of *M.epiphylla* (Fig. 2a-e), thereby corroborating the distinction. While the interpretations of So (2003) aimed to provide a comprehensive treatment of Asian *Metzgeria* species, the morphological and ecological specifics of these taxa suggest the need for separate recognition. Thus, a nuanced approach to classification that underscores the individual characteristics of each species is warranted, rather than broad categorisations that do not adequately capture their distinctions.

The distribution of *M.epiphylla* across different global regions remains an area of considerable uncertainty. Historical records by Kuwahara (1978b) and later clarifications by Piippo (1991) have acknowledged its presence beyond well-documented ranges. The supposed exclusions made by So (2002) regarding its presence in the Neotropics fail to provide definitive categorisations, necessitating further investigation and confirmation. Our findings align with earlier literature, advocating for a re-evaluation of its distribution and calling for more comprehensive studies to verify this species’ presence in regions such as El Salvador and Chile.

In conclusion, our detailed examination of morphological traits, along with an evaluation of historical taxonomic literature, comparisons between *M.epiphylla* and closely-related species underscore the genus’ morphological diversity, supporting the view that *M.epiphylla* represents a distinct species. The original figure by Evans (1923) for *M.epiphylla* emphasises key characteristics such as the cylindrical gemmae with long marginal spiny hairs, distinguishing it from similar species like *M.decipiens* and *M.fruticulosa*. Detailed morphological analyses, including features such as elongated sporophytes and leaf cross-sectional anatomy, further reinforce its unique status. For example, the characteristic blue-green dots on the leaf tips when dried differentiate *M.epiphylla* from species like *M.fruticulosa*. This examination advocates for a careful and nuanced classification approach within *Metzgeria*, emphasising the necessity for detailed morphological investigations to resolve taxonomic ambiguities effectively.

## Supplementary Material

XML Treatment for Metzgeria epiphylla
